# Intestinal Microbial Profiles of Wild Zobaidy (*Pampus argenteus*) Fish Characterized by 16S rRNA Next Generation Sequencing

**DOI:** 10.3390/cimb47110890

**Published:** 2025-10-28

**Authors:** Dina Albaijan, Dalal Albaijan, Abrar Akbar

**Affiliations:** 1 Health College, The Public Authority for Applied Education and Training, Kuwait City 13109, Kuwait; 2 Environment and Life Sciences Research Centre, Kuwait Institute for Scientific Research, Safat, Kuwait City 13109, Kuwait

**Keywords:** *Pampus argenteus*, Zobaidy, Silver pomfret, operational taxonomic units, genome diversity, metagenomics

## Abstract

*Pampus argenteus* (Zobaidy) is an important fish in Kuwait and the Gulf region due to its economic value in the fish industry. Analyzing the gut microbiome of Zobaidy can help determine the health status of the fish and its responses to environmental changes. In this study, we investigated the microbiome composition of the intestinal tract among seven wild-caught silver pomfret specimens sampled in the Arabian gulf. The 16S rRNA was sequenced using the Illumina platform; then, sequences were analyzed using several bioinformatics tools to identify the microbial diversity, taxonomical status, and functional aspects. The results were 5933 operational taxonomic units (OTUs) categorized into 35 phyla. *Proteobacteria*, *Firmicutes*, *Bacteroidota*, and *Actinobacterota* were most abundant in the Zobaidy and water samples. At the genus level, we found high relative abundances of Acinetobacter. The results indicated that *Lactococcus piscium*, *Enterococcus cecorum*, *Psychrobacter arenosus*, *Vagococcus salmoninarum*, and *Carnobacterium maltaromaticum* are the most commonly present species in the analyzed Zobaidy samples. A heatmap analysis indicated notable differences in the functional categories of intestinal microflora within the Zobaidy2 sample compared to other Zobaidy samples. It should be noted that microbiome studies can provide novel ways to enhance the overall welfare of fish, strengthen disease prevention, and increase sustainability in aquaculture production.

## 1. Introduction

Fish gut bacteria are increasingly crucial for overall health and host disease resistance [1]. Understanding the functions of these microbes in fish has thus become a focus of much attention. The Zobaidy fish (Pampus argenteus, also known as silver pomfret) holds economic significance in the fish industry of Kuwait and the Gulf region, as noted by AI-Husaini (2003) and Narges et al. (2011) [2,3]. The Zobaidy family (Stromateidae) is found across the Indo-Western Pacific region, including the eastern part of China, the western and southwestern Korean Peninsula, and West Asia, as mentioned by Pati (1982) and Gupta (2020) [4,5]. Zobaidy is Kuwait’s most dominant and commercially essential species, with a high economic value [6]. It is a native fish of the Gulf and is distributed throughout its waters. They move towards the north or south based on the currents’ flow, heading north during the summer and south during the winter [5]. Research from other areas suggests that a muddy–sandy bottom is crucial for creating the right habitat for finding food [7], with significant quantities of mature Zobaidy and post-larvae discovered in shallow coastal waters [4].

The distribution of the fish is influenced by many water quality factors, such as salinity, zooplankton, temperature, turbidity, and dissolved oxygen [8,9]. Zobaidy is a highly demanded fish in most Kuwaiti cuisines, but no management precautions have been taken to protect against fish stock depletion [10]. The establishment of microbial community data of the species is highly needed due to Kuwait’s high value and importance in food security, biodiversity, and species conservation. Because fish, in general, are a primary source of food in Kuwait, it is highly important to keep fisheries sustainable [11]. As there are few studies that address such issues, there is a demand for data to help achieve management goals.

In terms of the whole intestine and feeding type, the gut microbiota of fish at different developmental stages is diverse, and the greater the diversity, the more it contributes to the health and growth of fish [12]. The intestinal microbiota of many species comprises microbes with ecological functions carrying various activities, such as fermentation, methanogenesis, nitrogen fixation, nutrient cycling, and the metabolic activities required for energy flow in aquatic ecosystems [13,14]. These microbes, like bacteria, fungi, and algae, inhabit various intestinal tissues of fish, and aid in nutrient absorption and digestion, showing greater diversity [13,15]. Many studies have shown that the intestinal microbiome is closely related to the environmental conditions of local fish, and complex ecological factors eventually result in complex fish–microorganism interactions, evolution, and adaptation strategies [16,17]. The precise characteristics of the microbial community of Zobaidy fish in marine water have never been reported before. On the other hand, the intestinal microbiota of other fish is usually dominated by key bacterial phyla such as Proteobacteria and Firmicutes. Proteobacteria are often associated with nutrient cycling and host microbe signaling [17], while Firmicutes are associated with fermentative metabolism.

There has been a major interest in this fish and its aquaculture potential. Hence, molecular biology and next-generation sequencing techniques have been increasingly employed. The flexibility, sensitivity, and efficiency gained by using molecular biology protocols in studies of host–microbiome interactions and their influence on the host’s physiology and behavior have opened up extensive possibilities [18]. While our un-derstanding of the composition of the intestinal microbiota of Zobaidy fish has been improving, this research takes a step forward by surveying and characterizing the range of microbial profiles in wild-caught Zobaidy fish.

## 2. Materials and Methods

### 2.1. Sample Collection

The Zubaidi Fish were collected from the Souk Sharq Fish Market located in Kuwait City. Four Kuwaiti-caught and three Iranian-caught adult fish were purchased, all delivered alive on ice within two hours of landing. In addition, water samples were collected from the same area to serve as an environmental reference. Several attempts were made to obtain information about the exact coordinates of the catch by local fisherman, but this proved to be difficult. No such information could be obtained—only that the catch came from the North Arabian Gulf.

#### Fish Dissection

Aseptic techniques were in place during the preparation work of the fish dissections. First, the dissection started by using scissors to open the vent area of the fish, cutting upwards towards the stomach and up to the gills. New tools were used to cut and collect gill filaments, which were placed in the labeled Falcon tube. The intestines were then collected using separate scissors so as not to cross-contaminate. The intestine was cut into three parts—the upper, mid, and lower intestine—and were each placed into separate tubes. The Falcon tubes containing the samples were placed in a cooler box chilled with ice for transportation.

### 2.2. DNA Extraction and Amplicon Generation

The Zobaidy intestinal samples were considered for the study, and the DNA was extracted using a Magnetic Soil and Stool DNA Kit (TianGen biotech, Beijing, China, Catalog #: DP712). The 16S rRNA genes of distinct regions (16SV3-V4/16SV4-V5) were amplified. All PCR reactions were carried out using a Phusion^®^ High-Fidelity PCR Master Mix (15 μL) (New England Biolabs, Ipswich, MA, USA) with a 10 ng template DNA and 0.2 μM of primers mix. PCR condition includes an initial denaturation at 98 °C for 1 min, followed by 30 cycles of denaturation at 98 °C for 10 s, annealing at 50 °C for 30 s, and elongation at 72 °C for 30 s and 72 °C for 5 min.

### 2.3. Bioinformatics Analysis Pipeline

#### Data and Sequence Assembly

Paired-end reads were assigned to samples based on their unique barcodes. The process was performed through Python (V3.6.13), and adaptors were removed using cutadapt (V3.3). Paired-end reads were merged using FLASH (V1.2.11, http://ccb.jhu.edu/software/FLASH/ (accessed on 7 April 2022)) [19]. The generated sequences were called raw reads. Quality filtering on the raw reads was performed using the fastp (Version 0.23.1) software with default parametes (Q20 trimming, maximum 5 Ns, and minimum length 50 bp) to obtain high-quality clean reads [20].

### 2.4. Operational Taxonomic Units (OTU) Cluster and Species Annotation

Sequence analysis was performed by Uparse software (Uparse v7.0. 1001, http://drive5.com/uparse/ (accessed on 7 April 2022)) [21]. Sequences with ≥97% similarity were assigned to the same OTUs. In addition, the Silva Database (http://www.arbsilva.de/ (accessed on 7 April 2022)) [22] was also used to annotate taxonomic information. To study the phylogenetic relationship of different OTUs and the difference in the dominant species in different samples (groups), multiple sequence alignments were conducted using the MUSCLE software (Version 3.8.31, http://www.drive5.com/muscle/ (accessed on 7 April 2022)) [23]. Venn and Flower diagrams were generated in R using Perl and the SVG function.

### 2.5. Relative Abundance Estimations and Heatmap Generation

The top 10 taxa of each sample at each taxonomic rank (Phylum, Class, Order, Family, Genus, Species) were selected to plot the distribution histogram of relative abundance in Perl through the SVG function. ASVs were aligned with MAFFT (v7.505), masked, and a maximum-likelihood tree was inferred using FastTree 2.1.11 with the GTR + CAT model. The abundance information of the top 35 taxa of each sample at each taxonomic rank was used to draw the heatmap using R software (Version 4.0.3). In addition, the phylogenetic tree was generated in Perl with SVG function.

### 2.6. Alpha and Beta Diversity

Alpha diversity is used to analyze the complexity of sample diversity using Observed-species, Chao1, Shannon, Simpson, ACE, and good coverage. All these indices were calculated in our samples using QIIME2 (2023.2) and visualized using R software (Version 4.0.3). Rank abundance curves can reflect the richness and evenness of species with samples by observing the width and shape of the curves. It can be plotted by using the RColorBrewer package in R (Version 4.0.3). Insufficient sequencing number may lead to insufficient sample information. In contrast, excessive sequencing rounds can also result in an unnecessary increase in cost. Therefore, the determination of the proper amount of sequencing is essential. Plotting rarefaction curves provides the ability to discover the viability of the sequencing numbers. This is achieved through R with the Plyr library package (Version 1.8.9).

Beta diversity is used to evaluate differences between different samples in species complexity using both weighted and unweighted UniFrac in QIIME2 (2023.2). Cluster analysis was preceded by principal component analysis (PCA), which was applied to reduce the dimension of the original variables using the ade4 package (version 1.7-23) and ggplot2 package (version 3.3.x) in R software (Version 4.0.3). Principal Coordinate Analysis (PCoA) was performed to obtain principal coordinates and visualize complex, multidimensional data. A distance matrix of weighted or unweighted UniFrac among samples obtained before was transformed into a new set of orthogonal axes, by which the maximum variation factor is demonstrated by the first principal coordinate, the second maximum variation factor by the second principal coordinate, and so on. PCoA analysis was displayed by ade4 package and ggplot2 package in R software (Version 4.0.3). Non-metric multidimensional scaling (NMDS) was also implemented for data dimension reduction. Similar to the PCoA, NMDS uses the distance matrix, but it emphasizes the numerical rank instead. The distance between sample points on the diagram can only reflect the rank information rather than the numerical differences. NMDS analysis was implemented through R software with the ade4 and ggplot2 packages.

### 2.7. Function Prediction

To predict the functional profiles of microbial communities, PICRUSt2 (v2.5.1) was used [24]. First, the 16S rRNA copy numbers were normalized; then, the Kyoto Encyclopedia of Genes and Genomes (KEGG) Orthology (KO) database [25] was used to predict microbiota functions in each sample.

## 3. Results

### 3.1. Sequence Information and Taxonomic Assessment

**Figure 1:**
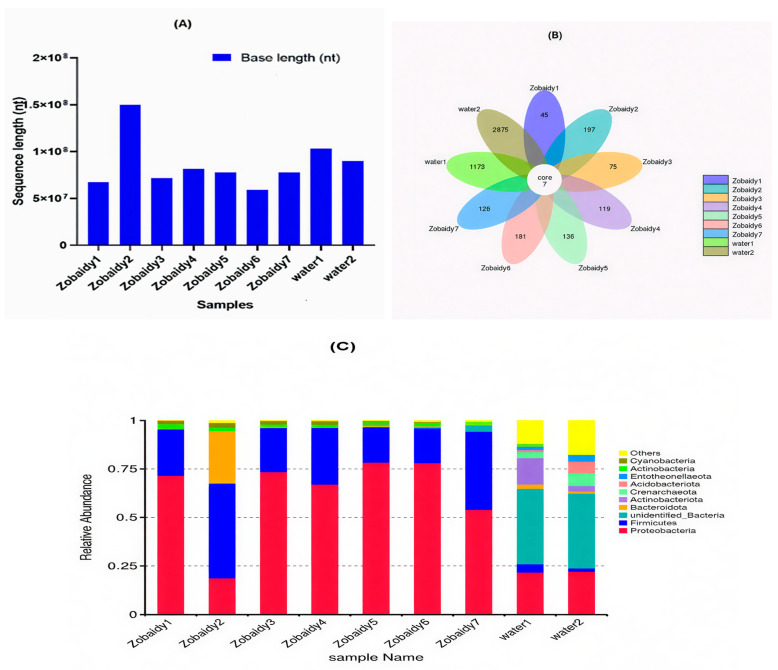
Read counts (**A**) and Venn diagrams of OTUs (**B**) and relative abundance of diverse microbes at phylum level (**C**) from each Zobaidy and water samples.

The Illumina MiSeq 16S rDNA sequencing data from seven Zobaidy and two water samples were analyzed to assess their microbial diversity. High-throughput sequencing of the V3–V4 region of the 16S rRNA gene was performed, followed by statistical analysis of the data from each sample. After rigorous quality filtration processes, a total of 7,785,545,567 read counts were obtained, with an average of 423.8 reads per sample (Figure 1A). The water samples exhibited a high average GC content of 56.10%, while the Zobaidy samples demonstrated a GC content of 52.06%.

A Venn diagram elucidated a total of 5933 Operational Taxonomic Units (OTUs), categorized into 35 phyla. The samples from healthy Zobaidy fish specimens (Zobaidy1 through Zobaidy7) and water samples (water1 and water2) comprised 45, 197, 75, 119, 136, 181, 126, 1173, and 2875 OTUs, respectively (Figure 1B). The mean OTU count was 125.57 ± 53.814 (*p* = 0.017) for Zobaidy samples and 2024 ± 1203.4 (*p* = 0.09) for water samples; the inter-group difference was statistically insignificant (*p* < 0.05). Figure 1C illustrates the top 10 microbial phyla among Zobaidy and water samples, predominantly including *Proteobacteria*, *Firmicutes*, *Bacteroidota*, *Actinobacterota*, *Crenarchaeota*, *Entotheonellaeota*, *Actinobacteria*, *Cyanobacteria*, unidentified bacteria, and others. Proteobacteria phylum was most abundant in Zobaidy samples, except in Zobaidy2, where Firmicutes predominated. Conversely, water samples exhibited a higher abundance of unidentified bacteria.

### 3.2. Alpha Diversity Among the Zobaidy and Water Samples

**Figure 2:**
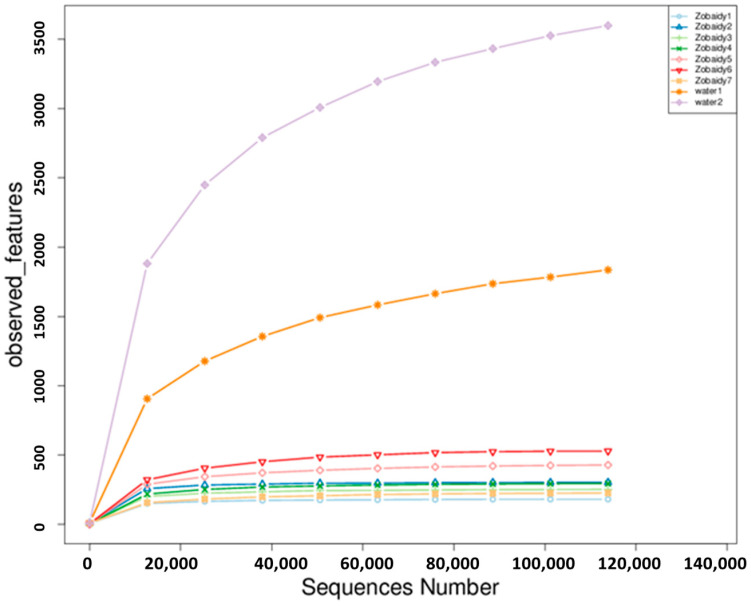
Refraction curve. Zobaidy1–7 represent the intestinal microbiota of seven healthy fish samples; water1–2 represent the microbiota in water sample.

The alpha diversity analysis was conducted to assess species diversity within samples, utilizing observed features and calculating Chao, pielou_e, Shannon, and Simpson indices based on OTU species and abundance. Observed features and Chao1 indices reflect species richness, quantifying OTU numbers. Shannon and Simpson indices were employed to evaluate community diversity, encompassing both species richness and evenness. Consequently, higher Shannon values and lower Simpson values indicate greater species diversity. Rarefaction curves reached asymptotes at maximum sequence numbers (Figure 2), suggesting adequate sequencing depth. Sequence integrity was evaluated using Good’s coverage, yielding 1 for Zobaidy samples and 0.99 for water samples. The coverage index ranged from 0.99 to 1, demonstrating that the sequences sufficiently represented sample species richness (Table 1). These comprehensive analyses provide a robust assessment of microbial community structure and diversity within the studied samples.

It is expected that the water sample shows greater microbial diversity than fish intestines, but such data allow for a quantitative ecological comparison and highlight the selective pressure and host-specific factors shaping the gut microbiome.

As illustrated in Table 1, the observed features and Chao indices exhibited significantly lower values in Zobaidy samples compared to water samples. Although individual fish samples such as Zobaidy6 exhibited relatively high observed features (528), the overall microbial diversity based on group level was lower, and this trend is supported by mean Chao1 and observed OUT value across groups. Furthermore, the Shannon index of the Zobaidy samples demonstrated a lower value than that of the water sample, and the Simpson index showed a similar reduction, albeit without statistical significance. The Chao1 indices, reflecting the species richness of the intestinal microbiota, were 317.97 ± 112.33 and 3153.667 ± 943.43 for Zobaidy fish and water samples, respectively. The Shannon and Simpson indices, indicative of microbiota diversity, were 3.915 ± 0.943 and 0.787 ± 0.10, respectively, for the Zobaidy fish group, and 7.961 ± 0.921 and 0.976 ± 0.009 for the water samples. Statistical analysis evaluated differences in alpha diversity indices between Zobaidy and water samples yielding significant difference (*p* < 0.05).

### 3.3. Beta Diversity Among the Zobaidy and Water Samples

**Figure 3:**
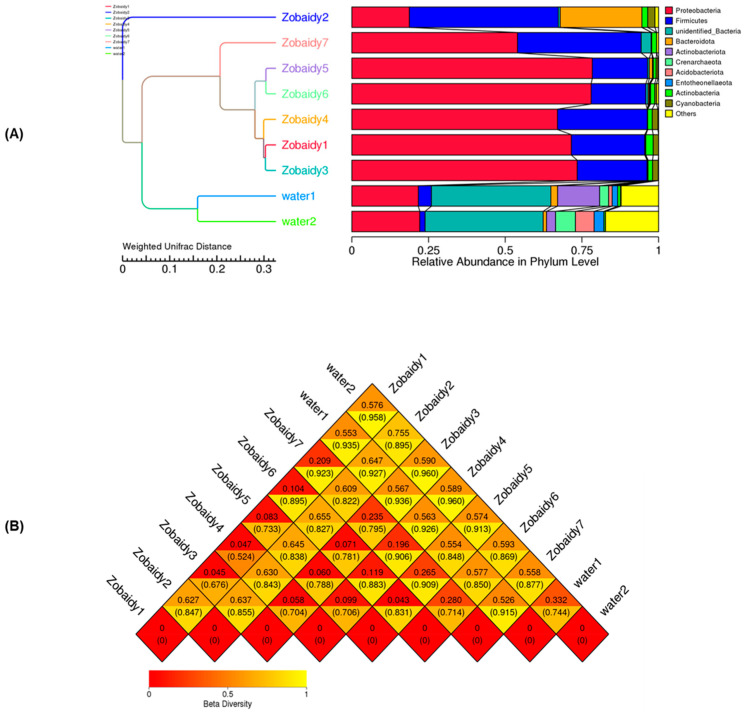
Beta diversity analysis estimated as relative abundance at the phylum level via the under unweighted pair group method, arithmetic means (UPGMA) clustering, and weighted UniFrac distance method (**A**), as well as the heatmap method (**B**). This figure complements the taxonomic abundance data by illustrating hierarchical relationships and compositional divergence.

To elucidate the intestinal microbiota composition of the Zobaidy and water samples, beta diversity analysis was employed. Unweighted pair group method with arithmetic means (UPGMA) clustering was utilized to account for abundance alterations and phylogenetic associations as indicators of beta diversity. The clustering analysis revealed that Zobaidy samples, with the exception of sample 2, formed a cohesive group, predominantly characterized by *Firmicutes*. Conversely, other samples exhibited a preponderance of *Proteobacteria*, followed by *Firmicutes*. The water samples demonstrated distinct microbial communities, indicative of their unique microbiota compositions (Figure 3A). In our study, a beta diversity heatmap displayed the highest distance value (0.960) for Zobaidy3 and water2, followed by Zobaidy4 and water2 samples, then followed by Zobaidy1 and water2 (0.958) (Figure 3B).

**Figure 4:**
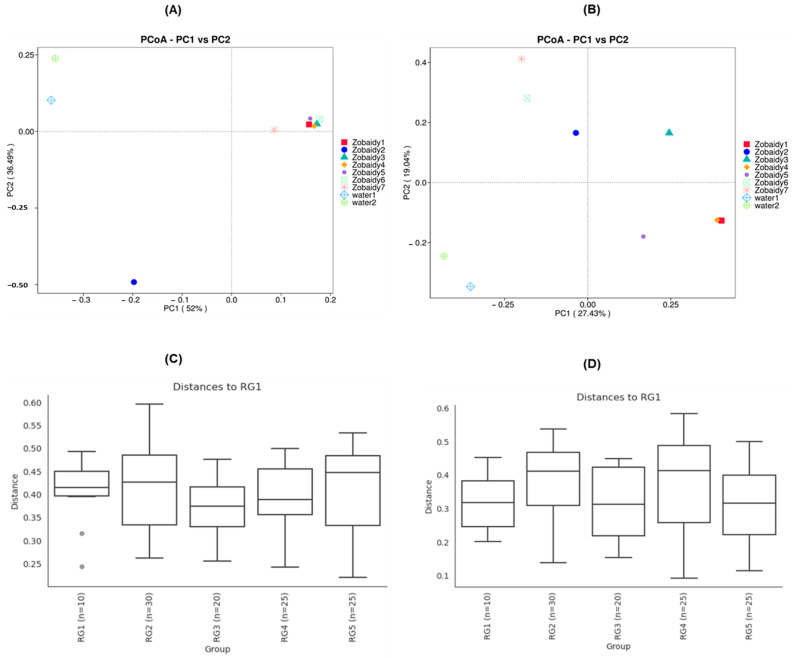
Principal coordinates analysis (PCoA) plots (**A**,**B**) and analysis of similarity (ANOSIM) (**C**,**D**) of beta diversity based on UniFrac metrics demonstrate statistically significant separation between the fish and water microbiome, highlighting the ecological and host-specific structuring of the microbial communities.

Principal coordinate analysis (PCoA) revealed significant variations among samples from different lakes (*p* < 0.001) (Figure 4). PCoA demonstrated that bacterial communities of Zobiady and water samples tended to cluster separately, with some overlap within Zobaidy samples, except for Zobaidy2 sample (Figure 4A,B), indicating distinct gut microbial communities for each group. Our findings showed that for unweighted UniFrac distance, PC1 and PC2 accounted for 52% and 36.49% of the total difference, respectively; while for weighted UniFrac distance, PC1 and PC2 accounted for 27.43% and 19.0% of the total disparity, respectively (Figure 4A,B). To corroborate this dissimilarity, we conducted an analysis of similarity (ANOSIM) on both unweighted and weighted UniFrac distance method. The ANOSIM results indicated no significant differences between the Zobaidy and water samples (unweighted R: −0.0001; *p* value = 0.532; weighted R: −0.034; *p* value = 0.641) (Figure 4C,D). Although principal coordinate analysis plots suggest some visual separation, the ANOSIM results demonstrate that these differences are not statistically robust.

### 3.4. Diversity and Composition of Intestinal Microbiota from Zobaidy Samples

Table 2 elucidates the microbial diversity spectrum across taxonomic levels in Zobaidy and water samples. Specimens such as Zobaidy2 and Zobaidy4 exhibit elevated diversity, potentially indicative of intricate microbiomes or conducive environmental parameters. Conversely, samples like Zobaidy6 and Zobaidy7 demonstrate reduced diversity, suggesting less-complex microbiomes or sampling from conditions supporting fewer microbial taxa. This dataset facilitates the further analysis of the environmental and biological factors influencing microbial diversity in *Pampus argenteus*. Among the aqueous samples, sample1 displayed superior diversity across all taxonomic strata. This phenomenon could be attributed to variable environmental factors between sampling locations, potentially offering insights into the microbial ecology of these habitats. Subsequent analysis could elucidate specific microbial communities or functional groups associated with the observed diversity patterns.

**Figure 5:**
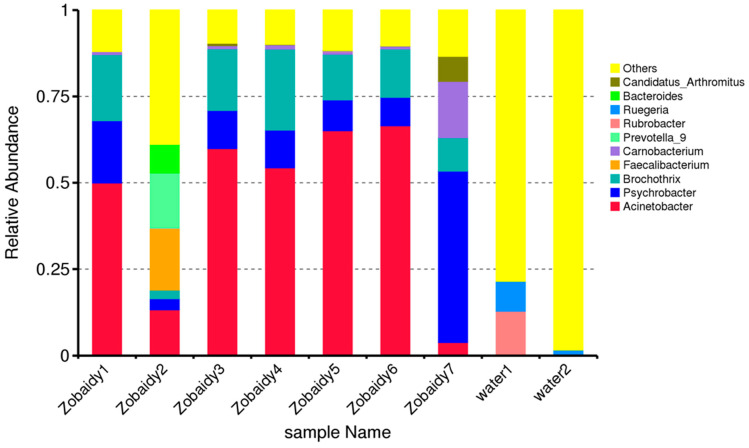
Relative abundance of diverse genera among various Zobaidy and water samples, providing higher taxonomic resolution than the phylum-level summaries in Figure 1C.

From the Zobaidy1 to Zobaidy7 samples, the predominant microbial community genera were identified as *Actinobacteria*, *Psychrobacter*, *Brochothrix*, *Faecalibacterium*, *Carnobacterium*, *Prevotella*_9, *Rubrobacter*, *Rugeria*, *Bacteroides*, and *Candidatus* (Figure 5). The Zobaidy2 sample exhibited diverse microflora, with *Faecalobacterium* being notably abundant. Conversely, Acinetobacter genera were more prevalent in all other samples. Water1 and water2 demonstrated entirely distinct microbes from various genera. Figure 5 illustrates the relative abundance of the top 10 species among the analyzed samples, including *Vagococcus salmoninarum*, *Lachnospiraceae bacterium* GAM79, *Alpha proteobacterium* DG1294, *Bacteroides caccae*, *Marvinbryantia geminata*, *Blautia obeum*, *Aliikanginella marina*, bacterium clone Anammox_9, *Lactococcus piscium*, and bacterium YC-ZSS-LKJ159.

### 3.5. Microbial Composition Among the Zobaidy and Water Samples

**Figure 6:**
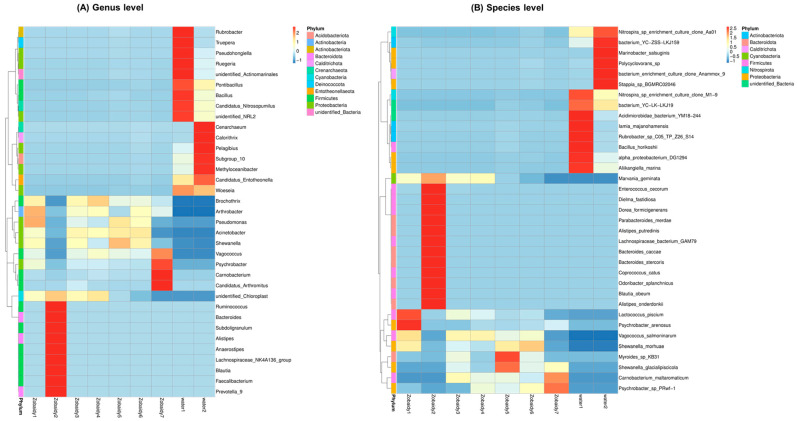
Heatmap of intestinal microbiota in Zobaidy and water samples. According to the species annotation and abundance information of all the samples at the (**A**) genus and (**B**) species level, we selected the top 35.

We analyzed the most abundant microbes (top 35) at the genus and species levels in the Zobaidy intestinal and water samples. The results indicated that *Brochothrix*, *Arthrobacter*, *Pseudomonas*, *Acinetobacter*, *Shewanella*, and *Vagococcus* are the most commonly present genera in the Zobaidy1, Zobaidy3, Zobaidy4, Zobaidy5, and Zobaidy6 samples; while the Zobaidy2, Zobaidy7, water1, and water2 samples have completely diverse genera (Figure 6A). Genera like *Ruminococcus*, *Bacteriodes*, *Alistipes*, *Anaerostipes*, *Lachnospiraceae* NK4A136, *Blautia*, *Faecalibacterium*, and *Prevotella* are more dominant in Zobaidy2. *Rubrobacter*, *Truepera*, *Pseudohonginella*, *Ruegeria*, *Pontibacillus*, *Bacillus*, and *Candidatus nitrosopumilus* are more prevalent in water1 sample; while in the water2 sample, *Cenarchaeum*, *Calorithrix*, *Pelagibius*, *Methylocaenibacter*, *Candidatus entotheonella*, and *Woeseia* are abundantly present (Figure 6A).

On further analysis, the heatmap of the top 35 at the species level (Figure 6B) revealed that the species *Lactococcus piscium*, *Psychrobacter arenosus*, *Vagococcus salmoninarum*, *Shewanella morhuae*, and *Marvania geminata* are abundantly present in Zobaidy1. Zobaidy2 gut microbiota showed entirely diverse microbes, including *Enterococcus cecorum*, *Dielma fastidiosa*, *Dorea formicigenerans*, *Parabacteroides merdae*, *Alistipes putredinis*, *Lachnospiraceae* bacterium GAM79, *Bacteroides caccae*, *Bacteroides stercoris*, *Coprococcus catus*, *Odoribacter splanchnicus*, *Blautia obeum*, *Alistipes onderdonkii*, and *Marvinbryantia geminata*are. In Zobaidy3 and Zobaidy6, *Vagococcus salmoninarum*, *Carnobacterium maltaromaticum*, *Myroides* sp. KB31, and *Marvinbryantia geminate* microbial species are present, whereas *M. geminate* was noticed to be absent in the Zobaidy6 sample. While Zobaidy4 mainly includes *M. geminate*, *V. salmoninarum*, *C. maltaromaticum*, and *Psychrobacter* sp., PRwf-1 are also present. Along with *V. salmoninarum* and *S. morhuae*, two new species were identified in the Zobaidy5 sample, which were abundantly present, namely, *Myroides* sp. KB31 and *S. glacialpiscicola*. Species such as *C. maltaromaticum* and *Psychrobacter* sp. PRwf-1 are the most abundantly present in Zobaidy7, followed by *S. glacialpiscicola*. The microbial flora of the environmental reference samples, water1 and water2, are entirely different and display significant diversity. Species such as *Nitrospira* sp. clone M1-9, *Acidimicrobidae bacterium* YM18-244, *Iamia majanohamensis*, *Rubrobacter* sp. C05-TP-Z26-S14, *Bacillus horikoshii*, *Alpha proteobacterium* DG1294, and *Aliikangiella marina* are most abundantly present in the water1 sample; while in the water2 sample, *Nitrospira* sp. clone Aa01, *Marinobacter salsuginis*, *Polycyclovorans* sp., and *Stappia* sp. BGMRC02046 are most abundantly present (Figure 6B). The presence of such diverse microbial flora suggests that certain mechanisms regulate different microbes in the Zobaidy intestines.

### 3.6. Functional Potential of Bacterial Community in Zobaidy Intestinal Samples Using PICRUSt

**Figure 7:**
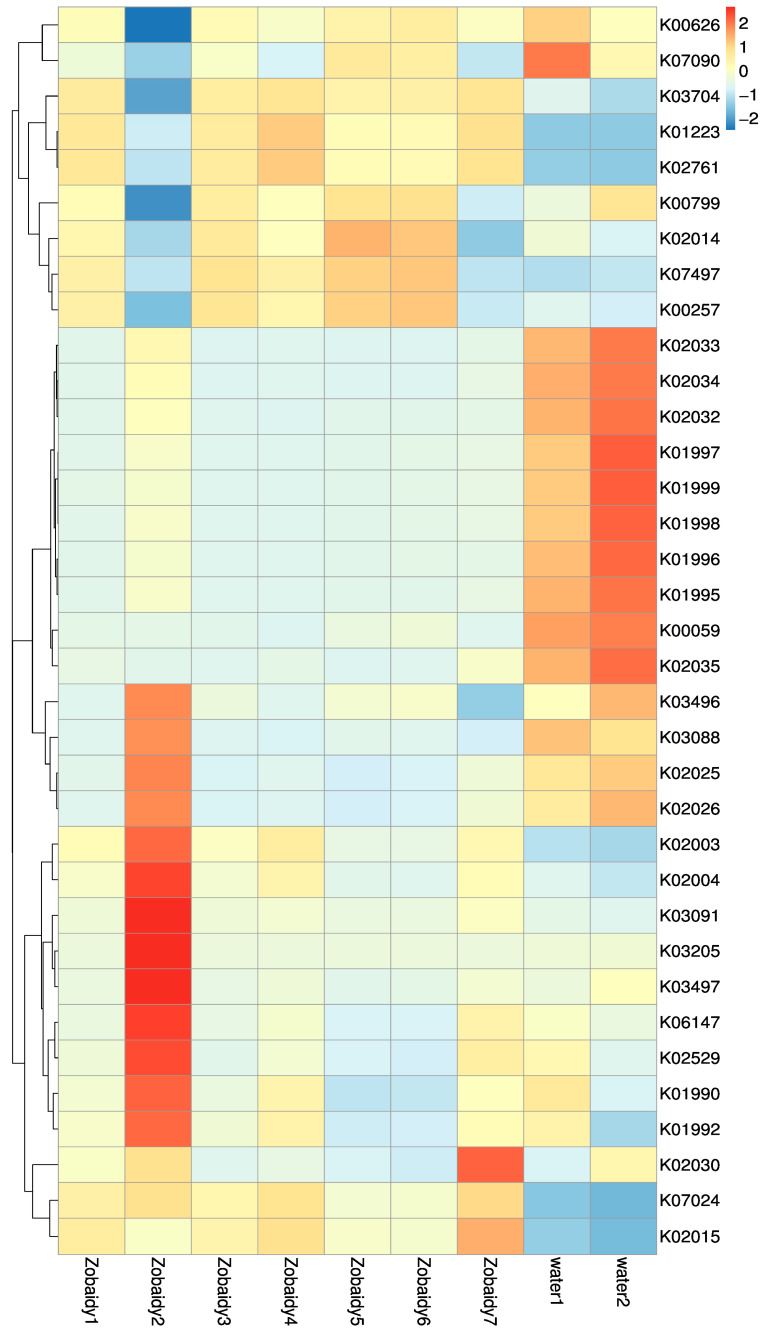
Heatmap showing the top 35 microbial functions of all Zobaidy and water samples at KEGG level.

To delve deeper into the interplay between gut microbiota and their host organisms, the PICRUSt tool was employed to predict the bacterial functional capacity across all samples, utilizing the KEGG database as a reference. A heat map, developed by the relative abundance of the top 35 KEGG pathways, revealed variations in metabolic pathways among different species, correlating with distinctions in core microbial families. Zobaidy1, Zobaidy3, Zobaidy4, Zobaidy5, Zobaidy6, and Zobaidy7 exhibited elevated levels of carbohydrate metabolism, specifically in starch and sucrose, fructose and mannose, galactose, and glycolysis/gluconeogenesis pathways (Figure 7, Table 3). In contrast, the Zobaidy2 sample demonstrated a greater expression of genes related to signaling and cellular processes, genetic information processing, and environmental information processing. The water1 and water2 contained functional attributes for peptide/nickel transport systems (cellular processes), branched-chain amino acid transport system permease, branched-chain amino acid transport system ATP-binding protein (environmental information processing), and fatty acid metabolism. The heatmap analysis indicated notable differences in the functional categories of intestinal microflora within the Zobaidy2 sample compared to other Zobaidy samples. This investigation provided deeper insights into the interconnection between the host’s trophic level and its metabolic capabilities.

## 4. Discussion

To the best of our knowledge we presented, this is the first 16S rRNA-based study on wild-caught Zobaidy (*Pampus argenteus*) in Kuwait, utilizing 16S rRNA next-generation sequencing techniques. The result identified 5933 OTUs across 35 phyla, where the predominant bacterial phyla identified are *Proteobacteria*, *Firmicutes*, and *Bacteroidota.* Notably, Acinetobacter and Psychrobacterbeing were consistently abundant across samples, suggesting their potential role as core microbiota in Zobaidy fish. Our comprehensive findings highlight the vital role that gut bacteria play in nutrient metabolism and disease resistance. The ecological uniqueness of the gut microbiota suggests that Silver Pomfret adapt their living conditions according to the host’s microhabitats, influencing geomicrobiology, with these microbiomes being regulated to optimize host health. Microbial diversity synthesis plays a crucial part in maintaining the host’s overall health. The structure of Zobaidy’s intestinal microbiota, alongside the quality and immune response within the fish intestine, holds significant potential for developing sustainable fish farming practices. Broadly, our study demonstrates the connection between fish gut microbial profiles and their physiological relevance and ecological roles in natural environments. Nonetheless, it remains uncertain as to how these microbial profiles could be practically applied.

The intestinal microbiota plays a crucial role in enhancing fish nutritional outcomes by aiding nutrient absorption and easing the biological demands on the host organism. Research has identified 15 primary phyla within the entire gut microbiome of rainbow trout, with Proteobacteria and Firmicutes being particularly prevalent in fish exhibiting superior nutrient uptake and metabolic efficiency. This indicates that a greater presence of these bacterial groups is indicative of enhanced fish health [26,27,28] Our investigation highlighted that Proteobacteria and Firmicutes, which potentially facilitate digestion and nutrient absorption, were the predominant phyla in the gut microbiota but were present as minor populations instead. The Venn diagram analysis in our study demonstrated that despite numerous shared operational taxonomic units (OTUs), distinct OTUs were present among various Zobaidy samples within a single habitat, suggesting that genetic makeup is likely the primary determinant influencing gut microbiota for hosts inhabiting identical geographical areas with similar environmental conditions. This conclusion is further corroborated by alpha and beta diversity assessments, which reveal marked disparities among the groups examined.

The primary genera, including *Actinobacteria*, *Psychrobacter*, and *Brochothrix*, exhibit capabilities for the detoxification of noxious compounds [29,30]. Beyond their involvement in nutrient digestion and absorption, the intestinal microbiota significantly influences the activation of immune responses in fish [31]. Notably, *Psychrobacter* is among the prevalent taxa in rapidly maturing grouper species, Epinephelus coioides. This genus is a crucial component of the marine ecosystem, particularly within the gastrointestinal tracts of teleost fish [32]. Zobaidy microbiomes had less overall diversity than other teleosts like rainbow trout and gilthead seabream [33,34], but they had the same amount of *Proteobacteria* and *Firmicutes.* However, the presence of *Carnobacterium maltaromaticum* and *Vagococcus salmoninarum* indicates niche-specific adaptation, potentially associated with the fish’s migratory patterns and Gulf habitat [35].

Furthermore, *Psychrobacter* predominates in the skin mucus of Atlantic salmon, *Salmo salar*, potentially indicating its role as a primary defense mechanism against bacterial infections [36,37]. Earlier research indicated that the primary phyla present in the stomach of the gilthead seabream, *Sparus aurata*, consisted of *Firmicutes*, *Proteobacteria*, and *Bacteroidetes* [38]. Contrarily, subsequent findings identified *Firmicutes*, *Proteobacteria*, and *Actinobacteria* as the predominant phyla [39]. Alterations in gut microbiota linked to captivity have been observed in both freshwater fish and other marine species, becoming a widely recognized phenomenon. Nevertheless, there remains a need for more comprehensive studies on gut microbiota in wild marine fish to establish a foundational comparison and enhance understanding of captive rearing impacts [40].

Firmicutes and Bacteroidota are capable of producing short-chain fatty acids (SCFAs) as byproducts of fermentation. These SCFAs have been demonstrated in mammals to influence immune system homeostasis, promoting IgA production and enhancing the differentiation of regulatory T cells within the small intestine’s lamina propria [41]. The KEGG analysis in our study revealed that most of the Zobaidy samples are involved in carbohydrate metabolism, cellular processes, environmental information processing, and genetic information processing. Within the scope of commensal microbiota, even the less prevalent major phyla have the potential to establish enduring, specific interactions with Zobaidy samples, as observed in our research. These interactions may confer protective benefits at the intestinal level through the secretion of antimicrobial compounds [42,43]. Collectively, the regulation of immune responses and the roles of commensal microbiota can bolster the probiotic advantages of a natural microbial community, thereby offering protection against inflammatory bowel disease (IBD) associated with dysbiosis [44,45].

Furthermore, the heterogeneity in the microbial taxonomic and functional profiles across Zobaidy samples (Figure 6 and Figure 7) reflects the dynamic nature of host-specific microbiota in wild fish. This variation likely arises from differences in environmental exposure, feeding behavior, and individual physiology. This diversity highlights the ecological plasticity of Zobaidy gut microbiota and suggests that even within a species, microbial colonization is shaped by multifactorial influences. The results provide insight into the adaptive microbial community inside the gut of wild fish.

Moreover, alterations in the fish intestinal microbiome composition, alongside the activation of host defense mechanisms, significantly influence the susceptibility and resilience to diseases mediated by the microbiota [46]. For instance, zebrafish display reduced resistance to bacterial infections when exposed to non-steroidal anti-inflammatory drug analogs conjugated with unnatural sugars, while the bacteria themselves remain unaffected [47,48]. Consequently, gut microbiota may have a direct impact on the disease resistance of fish, thereby influencing aquatic health overall [49]. Conversely, within our ecological environment, although the core microbiota associated with fish has been critically selected over extensive evolutionary periods, it continuously affects and shapes the host’s evolution, potentially through physiological trade-offs, especially concerning multi-stress resistance [50,51]. Consequently, symbiotic interactions on the host’s part may also play a role in fostering more resistant microbial strains, while indirectly influencing experimental selection frameworks [52]. Research conducted by Camara-Ruiz et al. indicated that the gut bacterium Shewanella putrefaciens, found in gilthead seabream and Senegalese sole, exhibits prebiotic properties [53]. It demonstrated in vitro antagonistic effects against known pathogens and showed the potential to boost the immune function and improve the stress resilience in fish. This implies that it might also lower vulnerability to microbial infections, as specifically observed in Zobaidy sample 1.

Future work will involve longitudinal sampling across seasons and geographic zones, which will help use to distinguish between environmental and host-driven microbiome variation. Moreover, experimental validation of candidate probiotic strains such as Psychrobacter and Carnobacterium could enhance aquaculture applications. The comprehensive data gathered during research could serve as a foundation for crafting breeding strategies aimed at enhancing the disease resistance of larvae and juvenile fish. The advantageous attributes of mature Zobaidy may be leveraged as a source of probiotics to enhance fish health by influencing the gut microbial balance. With insights into the microbial ecosystem of fish guts, tailored microbe-based cultures can be created for various larval phases. Additionally, we did not analyze dietary intake, which could also influence gut composition. But diets formulated with microbial elements can be designed to replicate the gut microbial environment of wild fish, optimizing performance in a financially sustainable and environmentally conscious manner [54]. Looking ahead, aquaculture technologies are likely to pivot towards employing probiotics instead of antibiotics, in favor of environmental care. Suppressing pathogenic microbes in fish guts through beneficial microbial compositions not only holds economic promise but also supports an industrial shift in fish feed formulation and conversion processes [1,55]. Our not being able to determine the exact location of the sample and the small sample size are the major limitations of this study, which may reduce the statistical power of group differences. However, this study provides valuable baseline data and should prove foundation to future research. Larger-scale studies with expanded sampling and geographic coverage are needed in order to validate these findings and explore population-level microbial dynamics.

## 5. Conclusions

This study, to the best of our knowledge, provides the first comprehensive identification of the gut microbiota of wild-caught *Pampus argenteus* fish in the region, revealing distinct microbial communities shaped by host-specific factors and environmental exposure. Fish microbiotas were dominated by phyla such as *Proteobacteria*, *Firmicutes*, *Bacteroidota*, and *Actinobacterota*. In addition, the presence of *Carnobacterium maltaromaticum* and *Vagococcus salmoninarum* indicates niche-specific adaptation, potentially associated with the fish’s migratory patterns and Arabian Gulf habitat, despite the limitations of this study in terms of sample traceability. The observed heterogeneity among fish samples underscores the dynamic nature of host–microbiome interaction. These findings lay the groundwork for future research into microbiome-based health analysis and probiotic application in regional aquaculture.

## Figures and Tables

**Table 1:** Alpha diversity indices and goods coverage of microbial 16S rRNA sequences from the gut of Zobaidy samples and water samples.

Sample Name	Observed Features	Chao1	pielou_e	Shannon	Simpson	Goods Coverage
Zobaidy1	181	182.2	0.480	3.596	0.791	1
Zobaidy2	303	303.6	0.727	5.995	0.963	1
Zobaidy3	252	256.5	0.442	3.523	0.746	1
Zobaidy4	294	295.44	0.418	3.430	0.752	1
Zobaidy5	428	432.529	0.401	3.506	0.694	1
Zobaidy6	528	528	0.369	3.334	0.672	1
Zobaidy7	226	227.565	0.514	4.020	0.894	1
water1	1836	2210.235	0.674	7.310	0.983	0.99
water2	3599	4097.099	0.729	8.612	0.97	0.99

**Table 2:** Taxonomical counts among diverse taxonomical units in Zobaidy and water samples.

Sample Name	Kingdom	Phylum	Class	Order	Family	Genus	Species
Zobaidy1	135,840	135,827	135,827	135,821	134,843	134,450	5499
Zobaidy2	335,344	332,893	332,893	332,800	326,896	307,624	35,175
Zobaidy3	141,180	141,042	141,042	141,037	140,403	140,244	5029
Zobaidy4	163,954	163,939	163,939	163,927	163,429	163,108	5664
Zobaidy5	154,155	153,960	153,956	153,905	153,694	152,676	4739
Zobaidy6	113,577	113,365	113,272	113,062	112,439	111,612	3032
Zobaidy7	128,669	128,191	128,191	128,187	127,901	123,541	1641
Water1	208,872	194,737	194,065	185,418	170,103	103,090	11,032
Water2	183,194	171,055	164,711	148,633	128,063	62,892	8841

**Table 3:** Metabolic pathways detected in the samples.

KEGG Code	Protein/Enzyme	Gene	Pathway
K00626	acetyl-CoA:acetyl-CoA C-acetyltransferase	*ACAT*, *atoB*	Carbohydrate metabolism
K07090	uncharacterized protein	-	Unclassified
K03704	cspA; cold shock protein	*cspA*	Transcription; genetic information processing
K01223	6-phospho-beta-glucosidase	*bglA*	Glycolysis/Gluconeogenesis
K02761	cellobiose PTS system EIIC component	*celB*; *chbC*	Starch and sucrose metabolism
K00799	glutathione S-transferase	*GST*; *gst*	Metabolism of other amino acids; pathways in cancer
K02014	iron complex outer membrane receptor protein	-	Signaling and cellular processes
K07497	putative transposase	-	DNA Replication and repair
K00257	acyl-ACP dehydrogenase	*mbtN*, *fadE14*	Unclassified
K02033	peptide/nickel transport system permease protein	*ABC.PE.P*	Cellular processes
K02034	peptide/nickel transport system permease protein	*ABC.PE.P1*	Cellular processes
K02032	peptide/nickel transport system ATP-binding protein	*ddpF*	Cellular processes
K01997	branched-chain amino acid transport system permease protein	*livH*	Environmental information processing
K01999	branched-chain amino acid transport system substrate-binding protein	*livK*	Environmental information processing
K01998	branched-chain amino acid transport system permease protein	*livM*	Environmental information processing
K01996	branched-chain amino acid transport system ATP-binding protein	*livF*	Environmental information processing
K01995	branched-chain amino acid transport system ATP-binding protein	*livG*	Environmental information processing
K00059	3-oxoacyl-[acyl-carrier protein] reductase	*fabG*, *OAR1*	Fatty acid biosynthesis
K02035	peptide/nickel transport system substrate-binding protein	*ABC.PE.S*	Cellular processes
K03496	chromosome partitioning protein	*parA*, *soj*;	Signaling and cellular processes
K03088	RNA polymerase sigma-70 factor	*rpoE*	Genetic information processing
K02025	multiple sugar transport system permease protein	*ABC.MS.P*	Signaling and cellular processes
K02026	multiple sugar transport system permease protein	*ABC.MS.P1*	Signaling and cellular processes
K02003	putative ABC transport system ATP-binding protein	*ABC.CD.A*	Signaling and cellular processes
K02004	putative ABC transport system permease protein	*ABC.CD.P*	Signaling and cellular processes
K03091	RNA polymerase sigma-E/F/G factor	*sigE_F_G*	Genetic information processing
K03205	type IV secretion system protein VirD4	*virD4*, *lvhD4*	Environmental information processing
K03497	ParB family transcriptional regulator	*parB*, *spo0J*	Genetic information processing
K06147	ATP-binding cassette	*ABCB-BAC*	Signaling and cellular processes
K02529	LacI family transcriptional regulator, galactose operon repressor	*galR*	Genetic information processing
K01990	ABC-2 type transport system ATP-binding protein	*ABC-2.A*	Signaling and cellular processes
K01992	ABC-2 type transport system permease protein	*ABC-2.P*	Signaling and cellular processes
K02030	polar amino acid transport system substrate-binding protein	*ABC.PA.S*	Signaling and cellular processes
K07024	sucrose-6-phosphatase	*SPP*	Starch and sucrose metabolism
K02015	iron complex transport system permease protein	*ABC.FEV.P*	Signaling and cellular processes

- not available.

## Data Availability

Data is contained within the article. And FastQ file (sequence files) are available upon request, Bioproject number: PRJNA1344668.
